# Expression of brain-derived neurotrophic factor in kidneys from normal and cyclosporine-treated rats

**DOI:** 10.1186/s12882-018-0852-2

**Published:** 2018-03-14

**Authors:** Yuan Sheng Tao, Shang Guo Piao, Ying Shun Jin, Ji Zhe Jin, Hai Lan Zheng, Hai Yan Zhao, Sun Woo Lim, Chul Woo Yang, Can Li

**Affiliations:** 1 Department of Nephrology, Yanbian University Hospital, #1327 Juzi St., Yanji, 133000 Jilin Province People’s Republic of China; 2 Health Examination Center, Yanbian University Hospital, #1327 Juzi St., Yanji, 133000 Jilin Province People’s Republic of China; 3 Transplant Research Center, Convergent Research Consortium for Immunologic Disease, The Catholic University of Korea, Seoul, South Korea; 4 Division of Nephrology, Department of Internal Medicine, Seoul St. Mary’s Hospital, The Catholic University of Korea, Seoul, South Korea

**Keywords:** Brain-derived neurotrophic factor, Tyrosine kinase, Cyclosporine A, Vasopressin, Urine concentration ability, Apoptosis

## Abstract

**Background:**

Accumulating evidence suggests that a decrease in brain-derived neurotrophic factor (BDNF) level induces a variety of psychiatric and neurological disorders. However, the expression and role of BDNF in the kidney have not been explored. The present study examined the expression of BDNF and tropomyosin-related kinase (Trk) receptors in an experimental model of chronic cyclosporine A (CsA) nephropathy.

**Methods:**

Sprague-Dawley rats on a salt-deplete diet were treated daily for four weeks with vehicle or CsA. Urine profiles, apoptotic cell death, oxidative stress (8-hydroxy-2′-deoxyguanosine, 8-OHdG), and expression of BDNF and Trk receptors (TrkB and TrkC) were compared between groups. The impact of vasopressin infusion on the urine-concentrating ability was examined by measuring the expression of aquaporin-2 (AQP-2) and BDNF and urine profiles in normal and CsA-treated rats.

**Results:**

Compared with the vehicle-treated rats, rats given CsA had enhanced urine volume and declined urine osmolality. Immunohistochemistry and immunoblotting showed that BDNF and Trk receptors were constitutively expressed in kidneys from vehicle-treated rats. This was confirmed by double immunofluorescent staining for Na-K-ATPase-α1, AQP-1, and AQP-2. By contrast, the expression of these factors decreased in kidneys from CsA-treated rats (BDNF: 51.1 ± 19.5% vs. 102.0 ± 30.3%, *p* < 0.01). Downregulation of BDNF was accompanied by impairment of urine osmolality, and this was reversed by exogenous infusion of vasopressin. Notably, the number of TUNEL-positive cells correlated negatively with BDNF expression and positively with urinary 8-OHdG excretion.

**Conclusions:**

BDNF is expressed in the collecting duct of the kidney and may be associated with urine-concentrating ability in an experimental model of chronic CsA-induced nephropathy. Our study provides a new avenue for further investigation of chronic CsA nephropathy.

## Background

Brain-derived neurotrophic factor (BDNF) belongs to the neurotrophin superfamily which promotes the proliferation, development, survival, and differentiation of neurons in the peripheral and central nervous systems [1]. BDNF is synthesized as pro-BDNF and is secreted as a mixture of pro-BDNF and mature BDNF. Mature BDNF binds to the tropomyosin-related kinase receptor B (tyrosine kinase B, TrkB) and thereby initiates phosphorylation through the mitogen-activated protein kinases, phosphatidylinositol 3-kinase, and phospholipase C-gamma signaling pathways, which ultimately accelerate protein synthesis, axonal growth, dendritic cell maturation, synaptic plasticity, and neuroprotective effect. By contrast, pro-BDNF binds preferentially to the p75 neurotrophin receptor which mediates apoptosis [2]. Low BDNF level or impairment of its signaling pathway is thought to be implicated in a variety of neuropsychiatric and neurological disorders. Although BDNF is strongly expressed in the neocortex, hippocampus, amygdale, and cerebellum of the central nervous system, it is also expressed in nonneuronal tissues including heart, retina, urethral sphincter, liver, airway smooth muscle, ovary, and fetal kidney [3–8]. Nevertheless, the role and expression of BDNF in the kidney remains underexplored.

Chronic cyclosporine A (CsA) nephropathy is a common complication observed during treatment for solid-organ transplantation and autoimmune diseases. This complication is manifested by afferent arteriolopathy, striped interstitial fibrosis, tubular atrophy, and progressive renal insufficiency [9]. CsA treatment also induces renal tubular injury manifested by polyuria, magnesium wasting, renal distal tubular acidosis, and hyperkalemia. Of these, impaired urinary-concentrating ability is a predominant feature of chronic CsA nephropathy in clinical practice [10]. Several factors, such as the renin-angiotensin system, nitric oxide, apoptosis, inflammatory mediators, innate immunity, transforming growth factor-β1 (TGF-β1), aquaporins, and urea transporters have been suggested as possible mediators of the evolution of chronic CsA nephropathy [9, 11]. However, the precise mechanism remains unclear.

Recent publications have delineated that calcineurin inhibitor (CsA or FK506) regulates the expression of the BDNF/TrkB pathway in the hippocampus and midbrain of astrocytes and that differential expression of BDNF/TrkB triggers different roles in the central nervous system, such as neurotoxicity or neuroprotection [12–14]. Considering the above mentioned background, we hypothesized that BDNF would be expressed in the kidney and may play a function because the renal tubules are vulnerable to CsA-induced insult. To test this hypothesis, this study was designed to assess the expression and localization of BDNF and Trk receptors in a well-established animal model of chronic CsA nephropathy.

## Methods

### Animals and drugs

Male Sprague-Dawley rats (Charles River, Wilmington, MA) weighing 200 to 220 g were placed in cages (Nalge Co., Rochester, NY) at 37 °C with a fixed light-dark cycle and were permitted food and water ad libitum. Starting 7 days before and continuing throughout the experimental period, rats were provided a low-salt diet (0.05% sodium, Teklad Premier, Madison, WI).

CsA (Novartis Pharma Ltd., Basle, Switzerland) was dissolved in olive oil to 15 mg/mL concentration. Exogenous 1-desamino-8-D-arginine vasopressin (DDAVP; Sigma, dissolved in 0.9% NaCl solution at 10 mM) was given via an implantable osmotic minipump.

### Study design

This protocol was approved by the Animal Care Committee of the Catholic University of Korea. Two animal studies were carried out.

#### Protocol1

The first experiment was conducted to examine the influence of CsA treatment on the expression of BDNF and Trk receptors. After acclimatization and a low-salt diet for 1 week, weight-matched rats were randomly divided into two groups and treated daily for 4 weeks as follows: 1) vehicle group (VH): rats were injected subcutaneously with olive oil (1 mL/kg); 2) CsA group: rats were injected subcutaneously with CsA (15 mg/kg). Each group contained 8 rats.

#### Protocol2

The second experiment was performed to examine the influence of exogenous vasopressin treatment on the expression of BDNF and AQP-2, and the urinary concentration in CsA-induced nephrotoxicity. The weight-matched rats were randomly divided into four groups and treated daily for 4 weeks as follows: 1) VH (*n* = 8); 2) VH + DDAVP (*n* = 8); 3) CsA (*n* = 8); 4) CsA + DDAVP (*n* = 8). The treatment protocol of olive oil and CsA was similar to that of protocol1. Exogenous DDAVP was administered for the last 7 study days as previously described [10, 15].

Under ketamine anesthesia, animals were euthanized at the end of study, and kidney specimens were withdrawn for further evaluations.

The investigators were blinded to the identity of the treatment group allocation and data analyses.

### Basic measurements

Body weight (BW) of pair-fed rats were recorded, and 24-h urine and blood samples for each rat were collected for measurement of urine osmolality (Fiske Associates, Norwood, MA), serum creatinine (Scr), blood urea nitrogen (BUN), and CsA concentration, as we have previously reported [16].

Systolic blood pressure (SBP) was measured with a tail manometer-tachometer system (BP-2000, Visitech system, Apex, NC). In brief, rats were placed into circular cases, and then SBP was detected using the tail-cuff system after rats were quiescent for 3–5 min. To obtain accurate measurements, three measurements for each rat were averaged.

### Histology

Kidney specimens were stained with Masson’s trichrome and hematoxylin. Evaluation of tubulointerstitial fibrosis (TIF) was performed as previously described [17, 18].

### Immunohistochemistry

Sections were incubated for BDNF (ab108383, Abcam’sRabMAb® technology, USA), TrkB (ab33655, Abcam’sRabMAb® technology USA), and TrkC (ab33656, Abcam’sRabMAb® technology, USA).

### Double immunofluorescence

Immunostaining of BDNF, TrkB and TrkC was performed using horseradish peroxidase conjugate as secondary antibody and 3,3′-diaminobenzidine as chromogen. Sections primary stained with BDNF, TrkB and TrkC were incubated with Na-K-ATPase-α1 (Abcam’sRabMAb®technology USA) antibody. Na-K-ATPase-α1 labeling was examined with fluorescein isothiocyanateY conjugated donkey anti-rabbit IgG antibody (Jackson ImmunoResearch Laboratories). The procedure of immunofluorescence for AQP1 (ab65837 Abcam’sRabMAb® technology USA), AQP2 (ab15116, Abcam’sRabMAb® technology USA), and Bcl-2 (Bioworld Co.,Minneapolis, MN, USA) was similar to that for Na-K-ATPase-α1. Images of the tissue sections were captured using a Carl Zeiss photomicroscope equipped with differential interference contrast optics and fluorescence imaging capabilities (Axio Imager M2; Carl Zeiss, Jena, Germany).

### Immunoblotting

Immunoblotting analyses were performed as previously described [17].BDNF, TrkB, TrkC, and AQP-2 were detected with specific antibodies. Densitometric analyses were referred to the VH group as 100%.

### Apoptotic cell death

In situ TdT-mediated dUTP-biotin nick end-labeling (TUNEL) assay was performed according to Apoptosis Detection Kit instruction manual (Intergen, NY). TUNEL-positive cells were auto-counted using a digital camera-based image analyzer at 20 nonoverlapped fields in each section.

### Examination of urinary BDNF and 8-Hydroxy-2′-Deoxyguanosine (8-OHdG)

Urinary total BDNF (R&D Systems, Inc., Minneapolis, MN, USA) level and oxidative stress biomarker 8-OHdG (Institute for the Control of Aging, Shizuoka, Japan) concentrations in serum and urine were examined using competitive enzyme-linked immunosorbent assay (ELISA).

### Statistical analysis

The data are presented as mean ± SEM. Comparisons between groups were accomplished using Student’s t-test for protocol1 and one-way ANOVA with post hoc Bonferroni test for protocol 2 (SPSS software version 21.0, Microsoft Corp.). Correlation coefficient analyses were used to examine the relationships among TUNEL-positive cells, BDNF expression, and urinary 8-OHdG. Significance was considered as *p* < 0.05.

## Results

### Expression of BDNF protein in VH- and CsA-treated rat kidneys

**Fig. 1:**
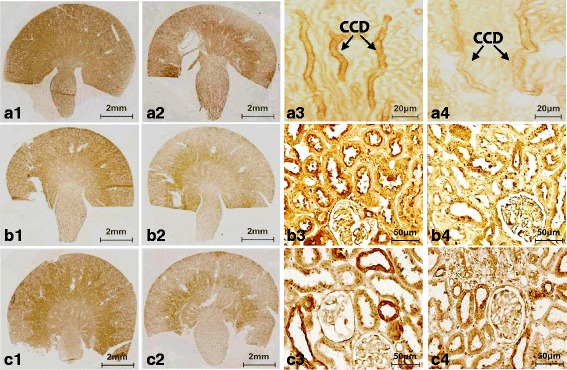
Representative photomicrographs of immunohistochemistry for BDNF (brain-derived neurotrophic factor), TrkB (tropomyosin-related kinase receptor **b**), and TrkC (tropomyosin-related kinasereceptor **c**) in the experimental groups. **a**1 and **a**3: BDNF in normal kidney; **a**2 and **a**4: BDNF in CsA-treated kidney. **b**1 and **b**3: TrkB in normal kidney; **b**2 and **b**4: TrkB in CsA-treated kidney.**c**1 and **c**3: TrkC in normal kidney; **c**2 and **c**4: TrkC in CsA-treated kidney. **a**1-**c**1 and **a**2-**c**2: original magnification X 40; **a**3-**c**3 and **a**4-**c**4: original magnification X 200; CCD: cortex collecting duct

**Fig. 2:**
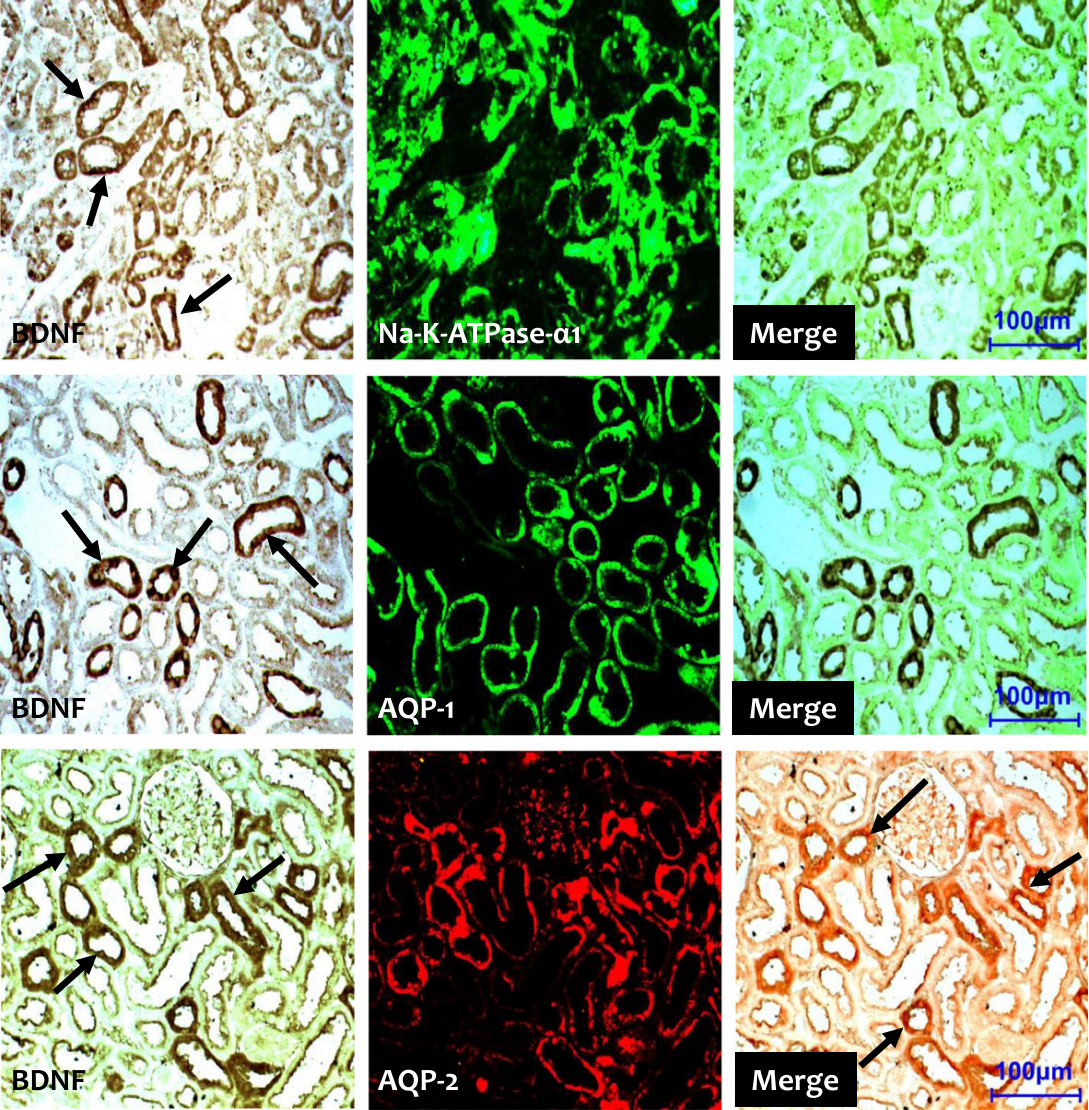
Representative photomicrographs of double immunofluorescent staining for BDNF (brain-derived neurotrophic factor) and Na-K-TAPase-α1 or AQP-1 (aquaporin-1) or AQP-2 (aquaporin-2) in normal rat kidneys. Original magnification: X 100

**Fig. 3:**
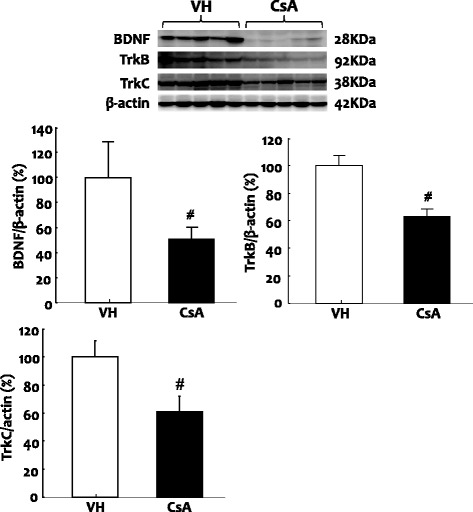
Representative photomicrographs of immunoblotting for BDNF, TrkB, TrkC in the treatment groups. Relative protein expression values are represented relative to the VH (vehicle) reference group, which is designated as 100% and are normalized to β-actin. ^*#*^*p* < 0.01 vs. VH

In the immunohistochemical analyses, BDNF immunoreactivity was detected in apical region of collecting duct cells in the cortex and medulla of normal rat kidneys (Fig. 1 a1 and a3). This staining pattern was confined to the principal and epithelial cells, as confirmed by double immunofluorescence staining for Na-K-ATPase-α1, AQP-1, and AQP2 (Fig. 2). Most other structures in the kidney were negative for BDNF. By contrast, BDNF protein expression was marked lower in CsA-treated rat kidneys compared with normal kidneys (Fig. 1 a2 and a4). Immunoblotting revealed that BDNF expression was about 50% lower after CsA treatment compared with VH treatment (Fig. 3; 51.1 ± 19.5% vs. 102.0 ± 30.3%, *p* < 0.05).

### Expression of Trk receptors protein in VH- and CsA-treated rat kidneys

**Fig. 4:**
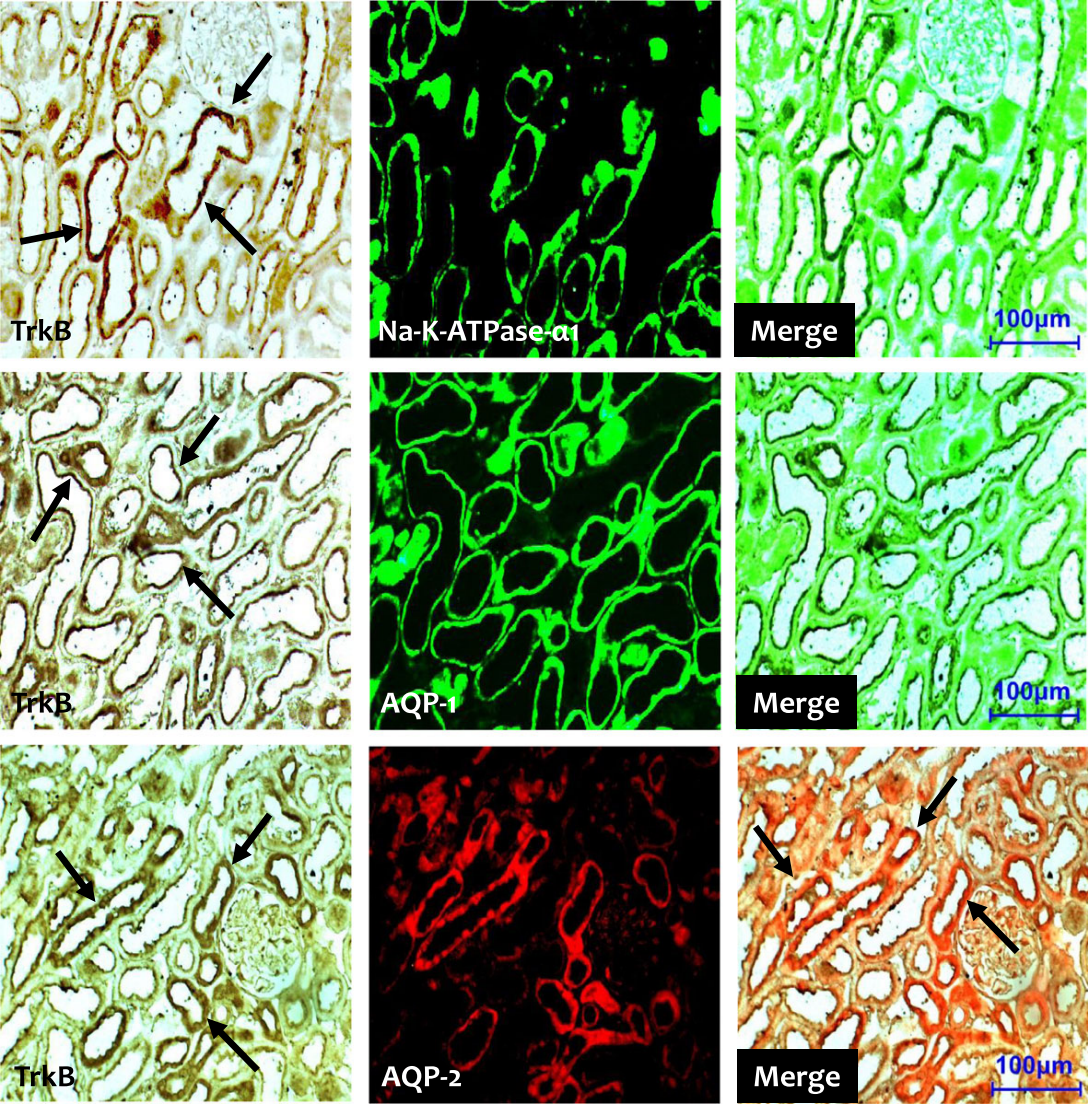
Representative photomicrographs of double immunofluorescent staining for TrkB and Na-K-TAPase-α1 or AQP-1 (aquaporin-1) or AQP-2 (aquaporin-2) in normal rat kidneys. Original magnification: X 100

**Fig. 5:**
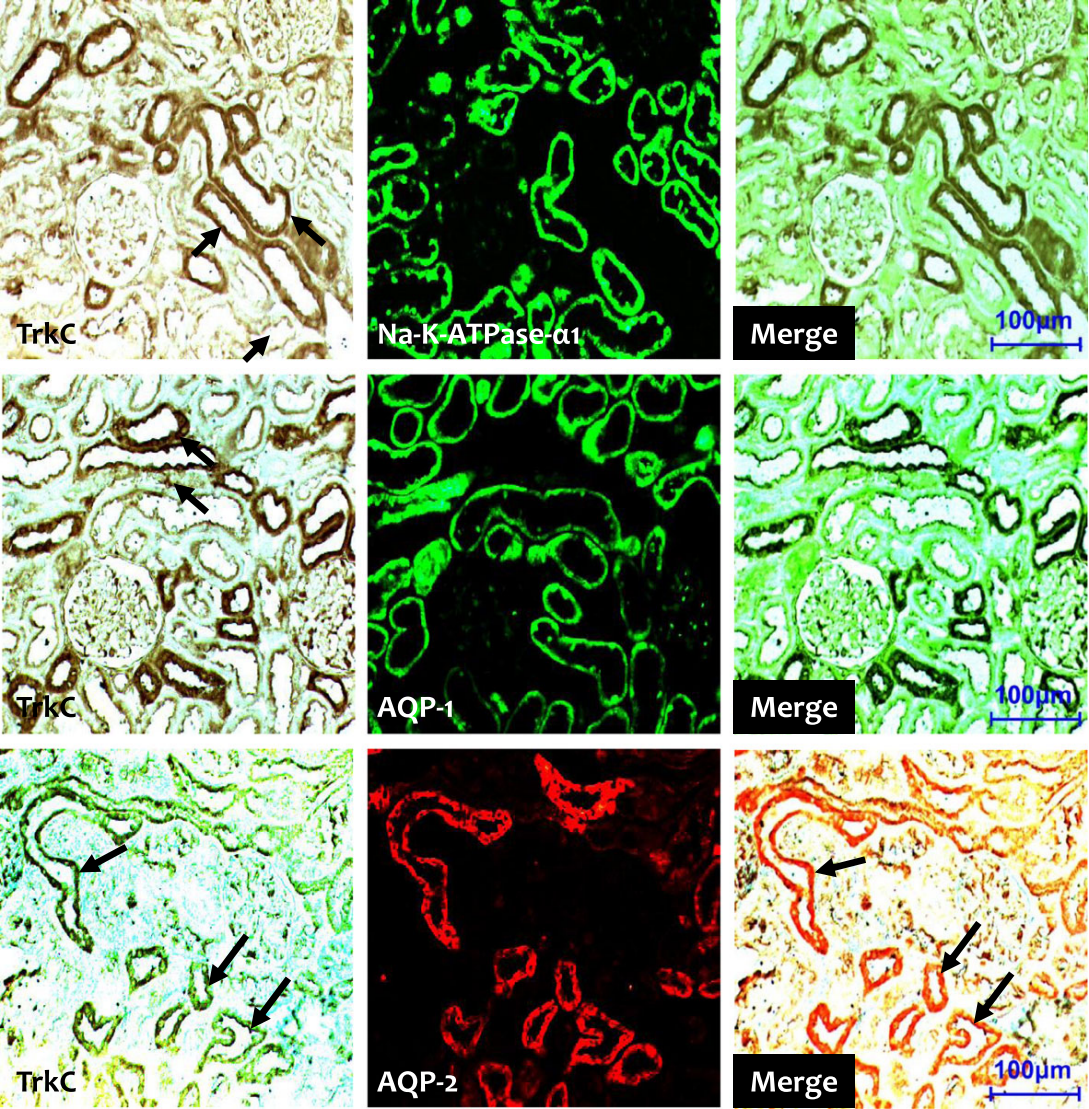
Representative photomicrographs of double immunofluorescent staining with TrkC (tropomyosin-related kinase receptor C) and Na-K-TAPase-α1, or AQP-1 (aquaporin-1), or AQP-2 (aquaporin-2) in normal rat kidneys. Original magnification: X 100

To explore the BDNF signal pathway, we used immunohistochemistry and immunoblotting to study the expression of TrkB and TrkC in the experimental groups. Immunohistochemistry showed that TrkB was expressed in the collecting duct of the cortex and medulla in normal rat kidneys (Fig. 1 b1 and b3), and that the expression was restricted to the apical region of epithelial tubules and the basolateral region of adjacent epithelial cells. Interestingly, double immunofluorescence staining of Na-K-ATPase-α1, AQP-1, and AQP2 showed that only TrkB staining merged with AQP-2 staining (Fig. 4). TrkB expression was markedly lower in the CsA-treated kidneys compared to that of VH treatment (Fig. 1 b2 and b4). The results of Immunohistochemistry were confirmed quantitatively by immunoblotting (Fig. 2; 61.7 ± 10.2% vs. 100.0 ± 12.4%, *p* < 0.05). Similar expression and localization patterns of TrkC were also shown in normal and CsA-treated rat kidneys (Figs. 1c, 2 and 5).

### Establishment of chronic CsA nephropathy

**Fig. 6:**
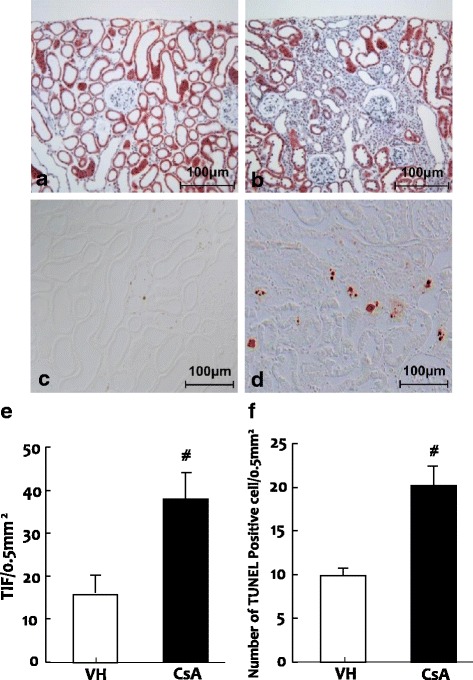
Trichrome staining (**a** and **b**) or TUNEL (TdT-mediated dUTP-biotin nick end-labeling assay; **c** and **d**) and semiquantitative analyses (**e** and **f**) in the VH (vehicle; **a** and **c**) and CsA (cyclosporine A; **b** and **d**) groups. **a** and **b**: original magnification X 100; **b** and **d**: original magnification X 200. ^*#*^*p* < 0.01 vs. VH

Table 1 outlines the basic examinations between groups. Rats treated with CsA displayed loss of BW (353.9 ± 12.3 g and 281.2 ± 11.1 g in VH- vs. CsA-treated rats, respectively; *p* < 0.05), renal insufficiency (Scr: 0.9 ± 0.3 mg/dL vs. 0.6 ± 0.1 mg/dL; BUN: 47.3 ± 11.4 mg/dL vs. 14.2 ± 2.4 mg/dL; both *p* < 0.05), and increased water intake (20.2 ± 4.5 mL vs. 15.3 ± 4.6 mL; *p* < 0.05) and urine volume (23.9 ± 6.2 mL vs. 10.3 ± 2.1 mL; *p* < 0.05). However, SBP did not differ significantly between groups (128.7 ± 11.3 mmHg vs. 124.2 ± 4.1 mmHg; *p* > 0.05). Histomorphological analysis showed that the long-term CsA treatment resulted in typically striped TIF (37.1 ± 3.2%/0.5mm^2^ vs. 10.1 ± 4.1%/0.5mm^2^; *p* < 0.05; Fig. 6b and e) and an increased number of TUNEL-positive cells in the renal tubules (Fig. 6d and f; 20.4 ± 5.2/0.5mm^2^ vs. 9.8 ± 2.1/0.5mm^2^; *p* < 0.05). These results confirmed the successful establishment of a rat model of chronic CsA nephropathy [10, 17, 19].

**Table 1:** Basic parameters

	VH (*n* = 8)	CsA (*n* = 8)
Body Weight (g)	353.9 ± 12.3	281 .2 ± 11.1^*^
Water Intake (mL)	15.3 ± 4.6	20.2 ± 4.5^*^
Urine Volume (mL)	10.3 ± 2.1	23.9 ± 6.2^*^
Urine BDNF (pg/mL)	0.06 ± 0.002	3.62 ± 1.02^*^
Urine 8-OHdG (ng/day)	303.9 ± 41.3	120.7 ± 27.0
Serum 8-OHdG (ng/mL)	82.9 ± 11.8	40.9 ± 7.5
BUN (mg/dL)	14.2 ± 2.4	47.3 ± 11.4^*^
Scr (mg/dL)	0.6 ± 0.1	0.9 ± 0.3^*^
SBP (mmHg)	124.2 ± 4.1	128.7 ± 11.3
CsA con. (ng/mL)	0 ± 0	1922.7 ± 22.3^*^

Values are means ± SEM. *VH* vehicle, *CsA* cyclosporine A, *BDNF* brain-derived neurotrophic factor, *8-OHdG* 8-hydroxy-2′-deoxyguanosine, *BUN* blood urea nitrogen, *Scr* serum creatinine, *SBP* systolic blood pressure. *p < 0.05 vs. VH

### Effect of CsA treatment on urine BDNF excretion

To determine whether suppression of BDNF expression in the kidney of CsA-treated rats is associated with changes in urinary BDNF excretion, we measured total urine BDNF concentration. Urine BDNF concentration was significantly increased in the CsA group when compared with the VH group (Table 1; 3.62 ± 1.020 pg/mL vs. 0.06 ± 0.002 pg/mL; *p* < 0.001). This result may be related to renal tubular dysfunction caused by CsA.

### Effect of CsA treatment on oxidative stress

We evaluated the effects of CsA on oxidative stress by measuring 8-OHdG in concentrations in the serum and urine, and comparing these between experimental groups. As shown in Table 1, serum and urine concentration were significantly higher in the CsA group than in the VH group (serum: 82.9 ± 11.8 ng/mL vs. 40.9 ± 7.5 ng/mL; urine: 303.9 ± 41.3 ng/day vs. 120.7 ± 27.0 ng/day; both *p* < 0.05). These findings suggest that long-term treatment of CsA induced oxidative stress injury by increasing 8-OHdG concentrations.

### Effect of DDAVP injection on BDNF expression and urine-concentrating ability in VH- and CsA-treated rat kidneys

**Fig. 7:**
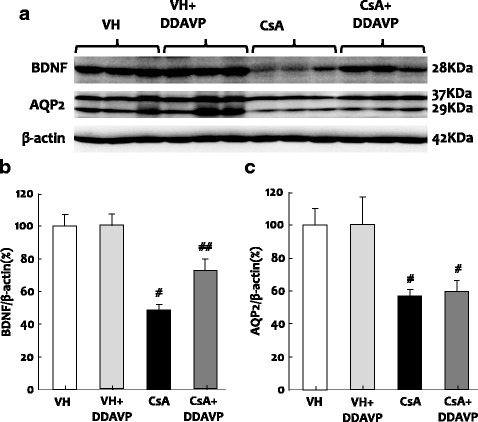
Immunoblotting (**a**) and desitometric analysis of BDNF (brain-derived neurotrophic factor; **b**) and AQP-2(aquaporin-2; **c**) expression in CsA (cyclosporineA)-treated rat kidneys after administration of DDAVP (1-desamino-8-D-arginine vasopressin). ^*#*^*p* < 0.01 vs. VH;^*##*^*p* < 0.01 vs. CsA

Given that BDNF and AQP-2 co-localized to the collecting duct system and that the suppression of BDNF expression by CsA is accompanied by impaired urinary concentration ability, we next examined the effect of DDAVP infusion on BDNF and AQP-2 expression and urine profiles. The expression of BDNF protein was significantly upregulated following DDAVP treatment in CsA-treated rat kidneys (BDNF: 76.9 ± 8% vs. 48.5 ± 10% in CsA- vs. VH-treated rats, *p* < 0.05), whereas AQP-2 expression was unchanged (Fig. 7; 60.5 ± 10.3% vs. 58.7 ± 7%; *p* > 0.05). Urine volume (Table 2; 9.9 ± 2.5 mL vs. 23.9 ± 2.8 mL; *p* < 0.05) and urinary osmolality (Table 2; 1356.7 ± 136.8 mosmoL/kgH2O vs. 515.7 ± 63.4 mosmoL/kgH2O; *p* < 0.05) were normalized after administration of DDAVP for 7 days.

**Table 2:** Urine volume and urine osmolality

	Urine Volume, mL/day		Urine Osmolality, vmosmoL/kgH_2_O	
	Day 21	Day 28	Day 21	Day 28
VH (*n* = 8)	12.8 ± 2.1	9.6 ± 1.7	1321.6 ± 10.9	1291.4 ± 8.7
VH + DDAVP (*n* = 8)	13.2 ± 2.5	4.9 ± 1.2^*^	1217.0 ± 87.4	3011.6 ± 117.4^*^
CsA (*n* = 8)	16.9 ± 3.2^*^	23.9 ± 2.8^*^	711.8 ± 116.4^*^	515.7 ± 63.4^*^
CsA + DDAVP (*n* = 8)	15.8 ± 3.4^*^	9.9 ± 2.5^#^	721.9 ± 172.7^*^	1356.7 ± 136.8^#^

Values are means ± SEM. VH: vehicle; CsA: cyclosporine A; DDAVP: 1-desamino-8-D-arginine vasopressin.*p < 0.05 vs. VH; ^#^*p* < 0.05 vs. CsA

### Association between apoptosis and BDNF protein expression and urinary 8-OHdG

**Fig. 8:**
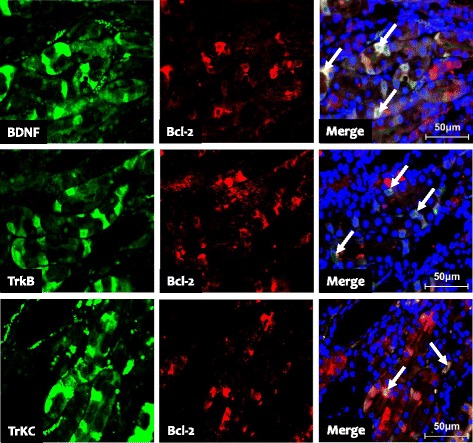
Double immunofluorescence with BDNF (brain-derived neurotrophic factor), TrkB (tropomyosin-related kinase receptor B), TrkC (tropomyosin-related kinase receptor C, green fluorescence) and Bcl-2 (B-cell lymphoma-2, red fluorescence) in kidneys from CsA (cyclosporineA)-treated rats. Immunofluorescence staining for BDNF, TrkB and TrkC serves as selective substrates for Bcl-2 (yellow fluorescence, arrows)

**Fig. 9:**
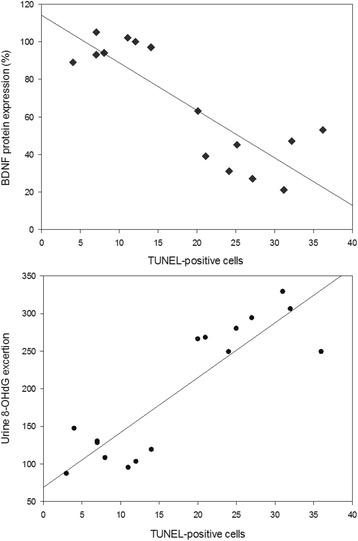
Relationship between the number of TUNEL (TdT-mediated dUTP-biotin nick end-labeling assay)-positive cells and BDNF (brain-derived neurotrophic factor) protein expression (*r* = − 0.866, *p* < 0.001) or urine 8-OHdG (8-hydroxy-2′-deoxyguanosine) excretion (*r* = 0.884, *p* < 0.001) in the treatment groups

The mechanisms by which CsA treatment decreases BDNF and its receptor expression in this study are unknown, but may be related to the increased apoptotic cell death seen in kidneys from CsA-treated rat. As shown in Fig. 8, double immunofluorescence with antibodies to BDNF, TrkB, TrkC, and Bcl-2 showed that BDNF, TrkB, and TrkC staining merged with Bcl-2 staining. Furthermore, TUNEL-positive cells correlated negatively with BDNF protein expression (Fig. 9: *r* = − 0.866, *p* < 0.001) and positively with urinary 8-OHdG (Fig. 9: *r* = 0.884, *p* < 0.001).

## Discussion

The present study clearly demonstrates that BDNF and Trk receptors (TrkB and TrkC) are constitutively expressed in the collecting duct of normal rat kidneys, whereas the expression is significantly lower in the kidneys of CsA-treated rats. Suppression of BDNF expression by CsA was paralleled by an increase in urine volume and a decrease in urinary osmolality. These effects were reversed after administration of DDAVP to the CsA-treated rats. Our findings suggest that the BDNF/Trk receptor system plays a role in the regulation of urine-concentrating capacity in chronic CsA nephropathy.

In addition to expression in the central nervous system, BDNF and Trk receptors are expressed in a wide range of nonneuronal tissues. However, few studies have examined the expression of BDNF and Trk receptors in the kidney. In the fetus, BDNF is expressed in the primitive glomeruli and primitive tubules, and Trk receptors (TrkB and TrkC) are expressed in the proximal and distal tubules and collecting tubule epithelial cells [8]. As development progresses, their expression becomes more prominent in the collecting duct system except for the glomeruli [20–22]. In the present study, double immunofluorescent staining showed that BDNF and Trk receptors were abundant in the collecting tubules of the cortex and medulla, but were confined to the principal cells and epithelial cells, whereas other structures were negative for BDNF and Trk receptors. Our findings are consistent with those of previous reports that found similar localization of BDNF and Trk receptors in the kidneys of teleosts, frogs, lizards, and humans [20–22], and suggest that the pattern of BDNF and Trk receptor expression changes during kidney development.

One interesting finding in this study is that CsA treatment significantly decreased BDNF expression, and this was accompanied by a decrease in TrkB and TrkC expression. The mechanism by which CsA suppresses BDNF and Trk receptor expression in this model may be direct or indirect. CsA has been shown to directly downregulate BDNF and Trk receptor expression in cultured SH-SY5Y cells in the hippocampus and midbrain, and that this may be related to the depressive symptoms of chronic CsA neurotoxicity [14]. In addition, CsA may indirectly decrease BDNF-Trk receptor expression by inducing apoptotic cell death. Amore et al. reported that CsA induces apoptotic cell death in cultured renal cells and murine tubular epithelial cells [23]. CsA is associated with hypoxia injury caused by afferent artery constriction, which ultimately results in oxidative stress [18]. It is accepted that oxidative stress stimulates apoptotic cell death, and that antioxidant therapy inhibits this program [24–26]. There is overwhelming evidence of a close relationship between oxidative stress and apoptosis in chronic CsA nephropathy [27, 28]. In the present study, treatment of rats with CsA increased the number of TUNEL-positive cells that are localized mainly to the proximal tubules, thick ascending limb, and collecting duct cells. Double immunofluorescence staining showed that BDNF and its receptors colocalized with Bcl-2 in the collecting duct cells in kidneys of CsA-treated rats. The existence of TUNEL-positive cells in collecting duct cells was ascertained by double immunolabeling for AQP-2 and the TUNEL assay, as previously reported by our laboratory [10]. Moreover, the number of TUNEL-positive cells correlated negatively with BDNF protein expression (*r* = − 0.866, *p* < 0.001) and positively with urinary 8-OHdG excretion (*r* = 0.884, *p* < 0.001). These findings suggest that loss of renal cells by apoptosis may partially account for the decreased BDNF expression in chronic CsA nephropathy.

BDNF functions as a neuroprotective agent against various neural insults through its role in neuronal cell proliferation, differentiation, and survival [1, 29]. These actions have been confirmed by studies of exogenous BDNF administration or BDNF-deficient animals, which have shown the neuroprotective effects of BDNF in the central nervous system [30–32]. However, the role of BDNF in the kidney is largely unknown. To identify its function in CsA toxicity, we infused exogenous DDAVP into CsA-treated rats. We found that BDNF expression was restored by DDAVP treatment and that this was accompanied by recovery of the urine-concentrating ability, as shown by improved urine osmolality and volume. The alterations in BDNF expression in this study lead us to speculate that the function of BDNF may be related to the regulation of urine-concentrating ability.

One potential limitation when interpreting the results of our study is the role of AQP-2 in urine-concentrating ability. AQP-2 is a member of a membrane protein family that plays a critical role in reabsorption of water in the kidney [33]. Therefore, changes in urine volume and osmolality during CsA or DDAVP treatment may arise from the actions of AQP-2 rather than BDNF. However, several items of evidence should be considered. First, we previously reported that AQP-2 was expressed in the apical region of collecting duct cells in normal rat kidneys, but that CsA treatment decreased AQP-2 protein expression only in the inner medulla of the collecting duct. However, AQP-2 protein expression within the cortex and outer medulla of the collecting duct did not differ between the VH- and CsA-treated groups [10, 34]. Second, BDNF expression was decreased in the CsA-treated rat kidneys throughout the collecting duct system in our current study (cortex, inner medulla, and outer medulla, Fig. 1b), and upregulation of BNDF expression by DDAVP was unaccompanied by restoration of AQP-2 expression. Third, BDNF may act as a neuromodulator [3, 34–36]. Using an animal model of bladder pain syndrome/interstitial cystitis, Frias et al. found that intrathecal injection of BDNF improved bladder function and relieved cyclophosphamide-induced cyctitis by modulating detrusor overactivity [33]. Murray et al. showed that increased BDNF expression in dorsal root ganglion cells of bladder afferents is involved in bladder innervations [36]. These findings suggest that BDNF plays a neuromodulatory role in urinary tract symptoms including urgency, frequency, and incontinence. Similar neuromodulatory effects of BDNF have been observed in congestive heart failure, insulin secretion after chronic exercise, and carbachol-induced contraction of intestinal longitudinal smooth muscle [3, 37, 38]. Thus, regulation of urine-concentrating ability, shown in this study, may contribute to the action of BDNF.

## Conclusions

This study demonstrates that BDNF and Trk receptors are expressed constitutively in the collecting duct of the normal kidney and that CsA decreases their expressions, which is accompanied by impaired urine-concentrating ability. Therefore, the function of BDNF may be associated with urine-concentrating capacity in the kidney. Additional studies are required to define this issue further.

## Abbreviations

TGF-β1: Transforming growth factor beta 1
8-OHdG: 8-hydroxy-2′-deoxyguanosine
AQP: Aquaporin
BDNF: Brain-derived neurotrophic factor
CsA: Cyclosporine A
DDAVP: 1-desamino-8-D-arginine vasopressin
Na-K-ATPase-α1: Natrium-Kalium-ATPase alpha1
TrK: Tropomyosin-related kinase
TUNEL: TdT-mediated dUTP nick end labeling
VH: Vehicle
